# A new variant of scaphoid reconstruction: Treatment of scaphoid non-union with avascular bone interponate and high compression screw (Synthes^®^)

**DOI:** 10.3205/iprs000066

**Published:** 2015-08-24

**Authors:** Christian Eder, Nina Schwab, Ariane Scheller, Björn Dirk Krapohl

**Affiliations:** 1 Centre for Musculosceletal Surgery, Charité – Medical University of Berlin, Germany; 2 Department of Plastic and Hand Surgery, St. Marien-Krankenhaus, Berlin, Germany

**Keywords:** scaphoid fracture, scaphoid non-union, compression screw, bone healing

## Abstract

Scaphoid fractures as frequently overseen injuries often result in scaphoid non-unions, that need to be treated to prevent carpal collapse and secondary cartilage damage. Vital bone tissue and compression of fracture and bone graft ends seem to be crucial in for ossification and final bone healing. In the present study we compare our results using a high compression screw (HCS Synthes^®^) to results in the literature using different kinds of internal fixation including compression screws of various types. We present 22 patients with scaphoid non-unions treated with a bone graft and a HCS Synthes^®^. We evaluated our post-operative results. The Manchester-Modified Disability of the Shoulder, Arm and Hand–Score (M-Dash) imposed with an average of 29.8 points (MD=29 / SD=9.46 / MIN=18 / MAX=48). None of the re-evaluated patients sorrowed for pain in rest. Five patients stated pain (ranging from 4 to 8 on numeric analogue scale) after heavy burden (e.g. boxing, weight lifting).In exploring the range of motion of the operated hand we deliver the following results: dorsal extension: average 72.73° (MD=80° / SD=17.23° / MIN=30° / MAX=85°), flexion: average 73.64° (MD=80° / SD=8.97° / MIN=60° / MAX=80°), ulnar deviation: average 39.09°, (MD=40° / SD=2.02° / MIN=35° / MAX=40°), radial deviation: average 29.09°, (MD=30° / SD=3.01° / MIN=20° / MAX=30°). Additionally a performance testing was conducted: fist clenching sign: complete without pain in 100%, pinch grip: complete in 100%, moderate pain in n=1 (8.33%), opposition digitus manus I–V complete in 100%, moderate pain n=2 (16.67%). Three patients with persisting fracture gap had a scaphoid bone fractured in the proximal third; one patient even with a very small proximal fragment. One persisting non-union was localized in the middle third (period between injury and operation = 5 years). In conclusion, our patients showed better healing rates compared to results presented in the literature. Non-unions localized in the proximal third of the scaphoid did not seem to benefit using this technique.

## Zusammenfassung

Kahnbeinbrüche werden häufig übersehen und führen zu Kahnbeinpseudarthrosen, die einer Therapie bedürfen, um den karpalen Kollaps und sekundären Knorpelschaden zu verhindern. Vitales Knochengewebe und Kompression der Frakturenden bzw. der Enden von Knocheninterponaten scheinen wesentlich für die Ossifikation und knöcherne Durchbauung zu sein. In der vorliegenden Studie vergleichen wir unsere Ergebnisse in der Pseudarthrosenversorgung mittels Knochenspan und der Hochkompressionschraube der Firma Synthes^®^ (HCS Synthes^®^) mit anderen Studien aus der Literatur. Wir stellen die Nachuntersuchungsergebnisse von 22 Patienten vor. Der Manchester-Modified Disability of the Shoulder, Arm and Hand–Score (M-Dash) lieferte durchschnittlich 29,8 Punkte (MD=29 / SD=9,46 / MIN=18 / MAX=48). Keiner der Patienten gab Ruheschmerzen an. Fünf Patienten beklagten Schmerzen in der Höhe von 4 bis 8 auf der numerischen Schmerzskala nach starker Belastung wie z.B. Boxen und Gewichtheben. Der Bewegungsumfang betrug an der betroffenen Hand: Extension: Mittelwert 72,73° (MD=80° / SD=17,23° / MIN=30° / MAX=85°), Flexion: Mittelwert 73,64° (MD=80° / SD=8,97° / MIN=60° / MAX=80°), Ulnarduktion: Mittelwert 39,09°, (MD=40° / SD=2,02° / MIN=35° / MAX=40°), Radialduktion: Mittelwert 29,09°, (MD=30° / SD=3,01° / MIN=20° / MAX=30°). Weitere Testergebnisse: Fist clenching sign: komplett ohne Schmerzen in 100%, Pinch grip: komplett in 100%, mäßige Schmerzen in n=1 (8,33%), Opposition digitus manus I–V komplett in 100%, mäßige Schmerzen n=2 (16,67%). Drei Patienten mit persistierendem Pseudarthrosenspalt hatten eine originäre Fraktur im proximalen Drittel, eine persistierende Pseudarthrose lag im mittleren Drittel bei einem Alter der initialen Pseudarthrose von 5 Jahren. Zusammenfassend zeigten unsere Patienten eine bessere knöcherne Heilungsrate als die Patienten der in der Literatur beschriebenen Studien. Lediglich bei den Pseudarthrosen im proximalen Drittel war unsere Methode nicht überlegen.

## Introduction

Scaphoid fractures are the most common fractures in the carpal region [1], [2], [3]. Although the treatment has been monitored and improved over many years, scaphoid non-unions as a consequence of insufficient bony healing are stated with 5% to 15% in literature [4], [5]. Autologous bone grafts, mostly harvested from the iliac crest, has become the gold standard for surgical treatment of scaphoid non-union/-pseudarthrosis [6], [7], [8], [9]. In further development and re-evaluation of patients’ outcome, a lot of different varieties and modifications have been described [1], [4], [10], [11], [12], [13], [14], [15], [16], [17]. The aim of this study is to evaluate the short- and long-term outcome of a modified treatment of scaphoid non-union by using the high compression screw (HCS Synthes^®^) and autologous avital (iliac bone, radius) interposition in comparison to other procedures.

## Material and methods

Between 2011 and 2013, 22 patients with scaphoid non-union were diagnosed and treated in the Centre for Musculosceletal Surgery at Charité Berlin using the below new compression screw. A persistent fracture gap six months after initial trauma was defined as scaphoid non-union according to the classification by Gupta et al. [18]. The patients were explored clinically and radiologically (either X-ray, computertomographic image or MRI) for diagnosing and preoperative planning. 

Retrospectively, patients’ medical recordings have been explored. Therefore age and gender, previous medical history, trauma history, operation technique (approaches, operation time, complications), pre- and postoperative radiological images, outcome up to three month after operation and healing rates were analyzed and summarized under short-term evaluation of the explained modified treatment. 

Furthermore, a long-term evaluation was performed. Patients under 18 years were excluded after guidance of the local ethics committee. Due to this constraint and patient’s non response, in conclusion a total of 12 patients could be re-evaluated. This long-term evaluation consisted of obtaining a general health questionnaire (for this a simplified SF-36 was chosen) and the score according to the Manchester-Modified Disability of the Arm, Shoulder and Hand [19]. Additionally, a clinical examination was performed with regard to the specific aspects: range of motion, sensory deficiencies, pain and strength. To objectify the strength regain, an investigation by using the hand-held dynamometer (JAMAR^®^) [20] was conducted. 

### Patients’ data

The 22 patients initially operated had an average age of 30.27 years (MD=25 / SD=13.5). The group includes 18 male (81.82%) and 4 female (18.18%) patients. 

Presented mechanisms of scaphoid fracturing:

- 1 case of punch-injury
- 3 cases of traffic accident
- 4 cases of sports injury
- 11 cases of fall (either by bike or in domesticity)
- 3 otherwise non-specified

One of the patients was treated conservatively in a different hospital and one patient was amended with high compression screw and a bony implant after presenting with implant failure due to a Matti-Russe-plasty operation which had been performed externally. The last one is going to be excluded in further analysis.

All of these 22 patients underwent surgery with the modified scaphoid reconstruction technique using an iliac crest bone graft combined with the high compression screw (HCS Synthes^®^). Concerning to the different non-union extents, 20 patients got autologous iliac crest interponates (90.9%), one got autologous distal radius interponates (4.55%) and one patient was treated without any bony interponate (4.55%). 

The Synthes^®^-HCS is a self-drilling canulated compression screw. It is supplied as a steel and a titanium screw, the later was used for our patients. Compared to other scaphoid compression screws it leads to shorter surgery due to a simplified surgical technique. It is also applicable for minimally invasive technique. Reverse-cutting flutes facilitate screw removal and prevent potential breaking of the implant. Two different thread lengths of the shaft car for an optimal implant for any fragment size. 

Immobilization (casting in short thumb cast) for 6 weeks was conducted. In case of inadequate healing (evaluation by surgeon) two more weeks of immobilization were added.

For long-term re-evaluation 2 patients had to be excluded because of being younger than 18 years. Furthermore, 7 patients were lost in follow-up and one patient has been excluded because of having undergone surgery in a different hospital before. In conclusion 12 patients were clinically examined again with a mean follow up of 26 months (MD=22 / SD=8.87 / MIN=15 / MAX=41). 

In 5 patients (41.67%) the fracture affected the dominant hand (always right hand) and in 7 cases (58.33%) the non-dominant hand was fractured (6 times right, 1 time left extremity).

As a retrospective study an evaluation of existing radiographic imaging during the clinical follow up until union was obtained. 

Generating radiological images in long-term evaluation was relinquished – due to no or just moderate clinical conspicuousness it was relinquished to generate new radiological images in long-term evaluation; the radiation exposure would not have been ethically and medically justifiable. 

All descriptive statistics were performed using average/mean (MD), standard deviation (SD), minimum (MIN) and maximum (MAX) and percentage. SPSS v19.0 (SPSS Inc., Chicago, Illinois) was used to create statistics.

## Results

### Short-term evaluation

Concerning to our short-term evaluation the data of 21 patients could have been analyzed. Average surgery time was 70.3 minutes (MD=64.5 / SD=19.3 / MIN=37 / MAX=107). A post-operative immobilization-time of 6.36 weeks in average (SD=0.79) was indicated. At the end of regular therapy and in the last indicated X-ray images (or CT if plain radiograph findings were suspicious) in average 3 months after operation, 17 patients (80.95%) imposed with incipient bony healing of scaphoid non-union in average 3 months after operation. Three patients with persisting fracture gap have had the scaphoid bone fractured in the proximal third; one patient even with a very small proximal fragment. One persisting non-union was localized in the middle third (period between injury and operation = 5 years).

### Long-term evaluation

In long-term evaluation, three patients with persisting fracture gap are included. One patient suffered from polyarthritis in both hands, additionally.

The results in the shortened SF-36 general health survey (self-reporting health status for the last four weeks) showed no significant constraints. Asking for the general health status in self-estimation one patient answered “very good”, seven patients answered “good”, two answered “bad”. No further significant results could be found in analyzing the SF-36.

The Manchester-Modified Disability of the Shoulder, Arm and Hand–Score (M-Dash) imposed with an average of 29.8 points (MD=29 / SD=9.46 / MIN=18 / MAX=48).

None of the re-evaluated patients sorrowed for pain in rest. Five patients stated pain (ranging from 4 to 8 on numeric analogue scale) after heavy burden (e.g. boxing, weight lifting).

Furthermore, two patients noticed dysesthesia after heavy burden und three in the morning, but irregularly an infrequently. 

In exploring the range of motion of the operated hand, the following results can be presented:

- dorsal extention: average 72.73°
  (MD=80° / SD=17.23° / MIN=30° / MAX=85°)
- flexion: average 73.64°
  (MD=80° / SD=8.97° / MIN=60° / MAX=80°)
- ulnar deviation: average 39.09°
  (MD=40° / SD=2.02° / MIN=35° / MAX=40°)
- radial deviation: average 29.09°
  (MD=30° / SD=3.01° / MIN=20° / MAX=30°)

Additionally a perfomance testing was conducted :

- fist clenching sign: complete without pain in 100%
- pinch grip: complete in 100%, moderate pain in n=1 (8.33%)
- opposition digitus manus I–V: complete in 100%, moderate pain n=2 (16.67%)

**Figure 1:**
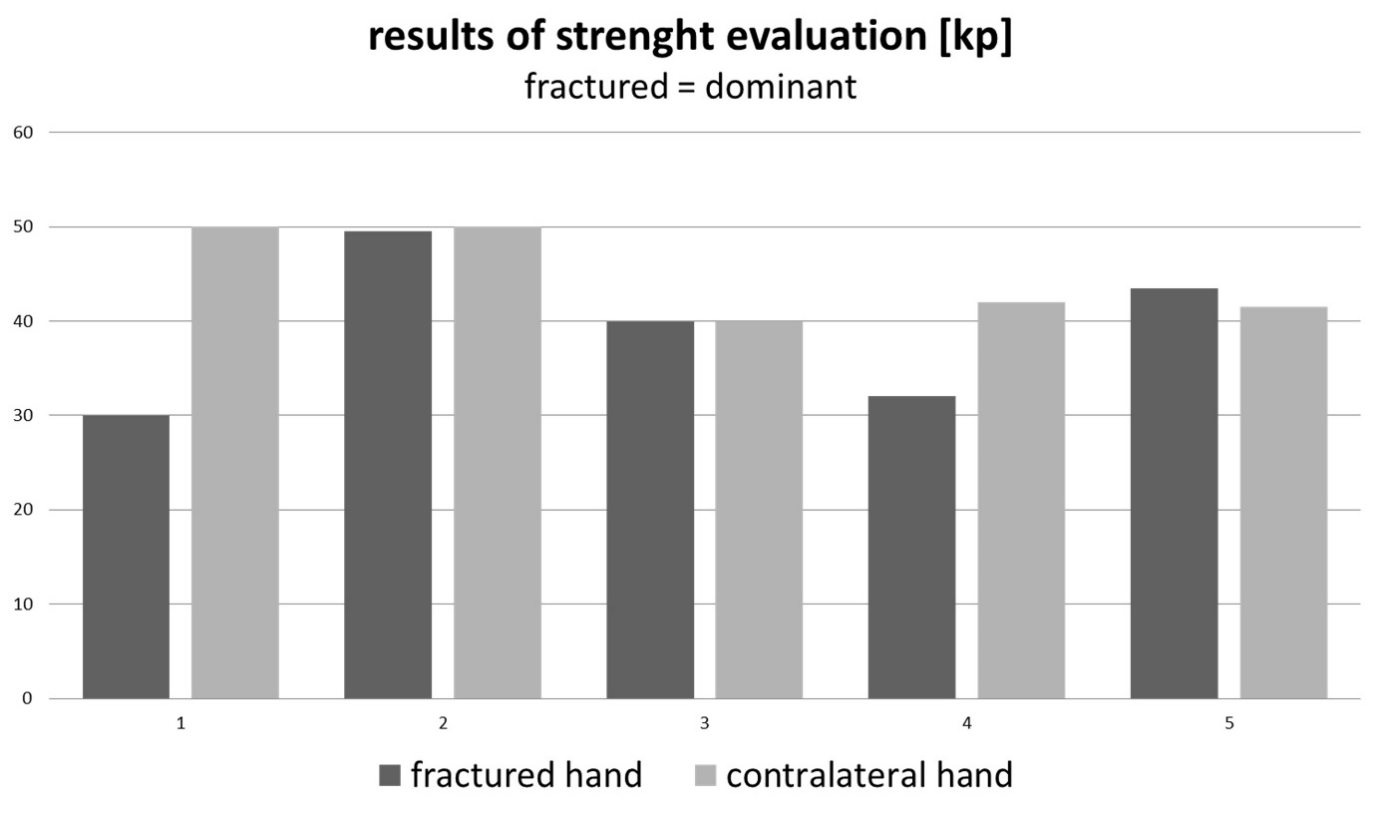
Difference in both hands in average 4.93 kp (MD=2 kp / SD=8.56 kp)

**Figure 2:**
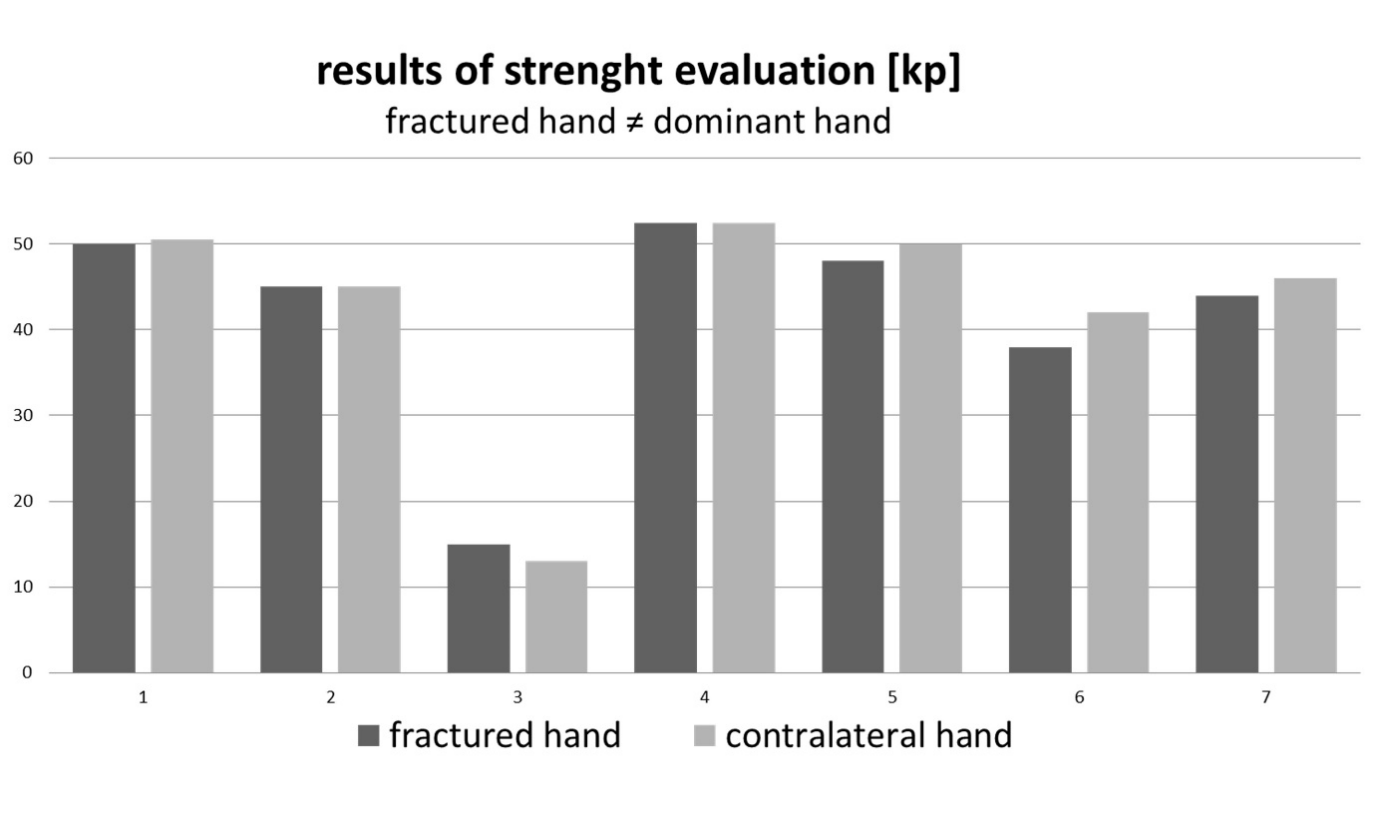
Difference in average 4.93 kp (MD=2 kp/ SD=9.4 kp)

Grip strength evaluation was performed by using the hand-held dynamometer. Patients were divided into two groups. Figure 1 (Fig. 1) showing patients with fractures of the dominant hand, Figure 2 (Fig. 2) patients who sustained fractures of the non-dominant hand.

**Figure 3:**
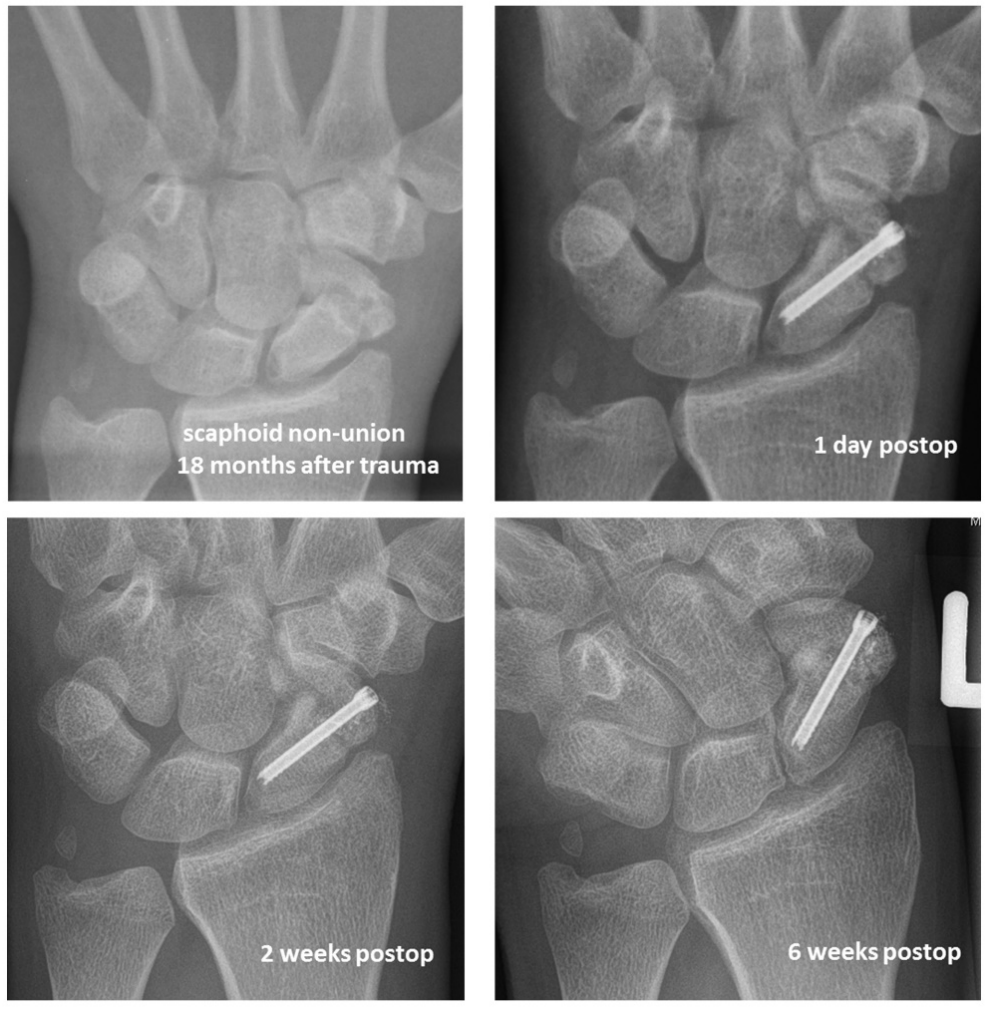
X-ray of an 18 months old scaphoid non-union of a 32 y.o. male treated with a bone graft from the iliac crest and an HCS (Synthes^®^), 6 week post-operative follow-up

Figure 3 (Fig. 3) shows an X-ray follow-up of an 18 months old scaphoid non-union successfully treated with a bone graft from the iliac crest and an HCS (Synthes^®^).

## Discussion

Treating scaphoid non-union with bone grafts and compression screws is widely accepted [6], [7], [21], [22]. Historically, this technique was performed without using any additional osteosynthesis material [6], [7].

Several modifications of the original Matti-Russe-plasty have been developed and described in the literature [1], [4], [10], [11], [12], [13], [14], [15], [16], [17], [23], [24], [25], [26], [27]. 

Based on a review of the literature we compared our results using the high compression screw (Synthes^®^) combined with avital autologous bone interponate with the most commonly used techniques:

- autologous cancellous bone interponate (distal radius) and Kirschner wire osteosynthesis
- Herbert screw osteosynthesis with or without avital bone interposition
- vascular pedicled bone graft and Herbert screw or Kirschner wire osteosynthesis

Park et al. described their technique using avital distal radius interponate with additional osteosynthesis by Kirschner wires. It resulted a general healing rate of 88.2% to 83.9%. The functional outcome was stated with 55° to 58.5° in dorsal extension and 58.2 to 59.5° in flexion of the wrist [4].

Using the Herbert screw osteosynthesis with or without avital bone interposition, Radford et al. stated a healing rate of scaphoid non-union (here defined as insufficient bone healing 6 month after injury, according to the Herbert and Fisher classification [28]) of 79% [29]. Furthermore, Maruthainar et al. described a scaphoid union after using Herbert screw and bone graft of 60% [30]. In both studies, the range of motion has not been published in detail. Merrell et al. and Munk et al. presented a general healing rate of 74% to 84% with the classical modified Matti-Russe-plasty (iliac bone and Herbert screw) [31], [32].

Werdin et al. examined their surgical technique of treating the proximal pole scaphoid non-union with vascularized bone graft and Herbert screw or Kirschner wire osteosynthesis respectively. A general union rate of 68.5% has been reported. Subdivided achievement ratios of 66.7% by performing osteosynthesis with Herbert screw and 69.4% with K-wire were presented [33]. In further clinical trials, healing rates after treatment with different vascularized bone grafts range from 27% to 100% [29], [30], [31], [32], [33], [34], [35], [36]. In the literature there has been a discussion regarding the preferred localization for the bone graft [32], [33], [34], [35], [36], [37], [38], [39]. 

It has to be mentioned that results and analysis of the screw versus K-wire osteosynthesis in treating scaphoid non-union has been discussed with conflicting results in the recent literature. On the one hand Jones et al. described no significant difference in these both procedures, on the other hand Merrell et al. stated that Herbert screw fixation is superior comparing to Kirschner wire fixation [31], [40]. 

In comparison to Park’s et al. results in post-operative range of motion out-comes (using K-wire fixation), the HCS (Synthes^®^) variant seems to be more successful in achieving functional recovery. Our results showing a union rate of 85.7% and good functional outcome suggest that using a HCS screw (Synthes^®^) instead of the common Herbert screw is a successful alternative method treating scaphoid non-union. However, it has to be noted that the high compression screw osteosynthesis failed in three patients having a scaphoid non-union in the proximal third. This fracture localization is known as a further risk factor for non satisfying treatment [33]. Taleisnik et al. described the anatomically critical blood supply of the proximal pole of scaphoid bone by the distal radial and lateral volar artery groups [41]. Therefore a high risk of avascular necrosis and a non-union rate up to 30% has been described in literature [4], [33]. The results of our study show that the modified variation of scaphoid reconstruction with HCS (Synthes^®^) is not able to guarantee higher chances for reaching bony union in proximal scaphoid non-unions.

It has to be pointed out that both the surgical technique and the duration of post-operative immobilization affect functional recovery. The here presented procedure has been performed by the same surgeon using a palmar approach. The post-operative treatment was a combined immobilization with a short thumb cast (6.36 weeks in average) and subsequent physiotherapy. The long-term evaluation showed a range of motion of 72.73° - 0 - 73.64° (dorsal extension – palmar flexion) and an ulnar deviation of 39.09° to radial deviation of 29.09° (all average). 

In comparison to Hirches’s et al. postoperative treatment with an average immobilization time of 12 weeks the results of this study showed an additional range of motion of 45.37° regarding extension and flexion (improvement of 31%) [42]. Furthermore, Park et al. performed a period of post-operation immobilization of 7.5 to 8 weeks and reached a healing rate of 88.2% to 83.9% and functional outcome with 55° to 58.5° in dorsal extension and 58.2 to 59.5° in flexion [4]. 

We assume, that shorter immobilization may lead to better functional recovery although other factors may determine functional outcome as well. Further studies have to be performed to verify this hypothesis. 

The literature presented several risk factors regarding bony healing treating scaphoid non-union; the most common ones are [4], [12], [43]:

- patient’s age
- time until treatment
- former operations
- blood supply in proximal pole
- smoking

In our study we could show that a persistent non-union of 20 years could be treated successfully, suggesting that time might not be the limiting factor regarding bony union. 

We have to admit that this study has some limitations: 7 patients were lost in follow-up (31.82%), a restricted power of analysis due to small and not representative patient population has to be noted and in our presented study the high compression screw (Synthes^®^) was used exceptionless, therefore all comparisons could just have been performed by reviewing actual literature.

## Conclusion

The here presented alternative of the original modified Matti-Russe-plasty showed better healing rates compared to results presented in the literature. Non-unions localized in the proximal third of the scaphoid did not seem to benefit using this technique.

## Notes

### Competing interests

The authors declare that they have no competing interests.
